# Membrane Vesicles of Toxigenic *Clostridioides difficile* Affect the Metabolism of Liver HepG2 Cells

**DOI:** 10.3390/antiox12040818

**Published:** 2023-03-27

**Authors:** Estefanía Caballano-Infantes, Ailec Ho-Plágaro, Carlos López-Gómez, Flores Martín-Reyes, Francisca Rodríguez-Pacheco, Bernard Taminiau, Georges Daube, Lourdes Garrido-Sánchez, Guillermo Alcaín-Martínez, Raúl J. Andrade, Miren García-Cortés, M. Isabel Lucena, Eduardo García-Fuentes, Cristina Rodríguez-Díaz

**Affiliations:** 1 Department of Regeneration and Cell Therapy Andalusian, Center for Molecular Biology and Regenerative Medicine (CABIMER), University of Pablo de Olavide-University of Seville-CSIC, Junta de Andalucía, 41092 Seville, Spain; 2 Instituto de Investigación Biomédica de Málaga y Plataforma en Nanomedicina, IBIMA Plataforma BIONAND, 29010 Málaga, Spain; 3 UGC de Aparato Digestivo, Hospital Universitario Virgen de la Victoria, 29010 Málaga, Spain; 4 Fundamental and Applied Research for Animals & Health (FARAH), Department of Food Microbiology, Faculty of Veterinary Medicine, University of Liège, 4000 Liège, Belgium; 5 UGC de Endocrinología y Nutrición, Hospital Universitario Virgen de la Victoria, 29010 Málaga, Spain; 6 CIBER de Fisiopatología de la Obesidad y Nutrición, Instituto de Salud Carlos III, 28029 Madrid, Spain; 7 Departamento de Medicina, Facultad de Medicina, Universidad de Málaga, 29010 Málaga, Spain; 8 CIBER de Enfermedades Hepáticas y Digestivas, Instituto de Salud Carlos III, 28029 Madrid, Spain; 9 Servicio de Farmacología Clínica, Hospital Universitario Virgen de la Victoria, Departamento de Farmacología, Facultad de Medicina, Universidad de Málaga, 29010 Málaga, Spain; 10 UICEC IBIMA, Plataforma SCReN (Spanish Clinical Research Network), Servicio de Farmacología Clínica, Hospital Universitario Virgen de la Victoria, Universidad de Málaga, 29010 Málaga, Spain

**Keywords:** *Clostridioides difficile*, extracellular vesicles, metagenomic, liver diseases, metabolic pathways

## Abstract

*Clostridioides difficile* infection (CDI) appears to be associated with different liver diseases. *C. difficile* secretes membrane vesicles (MVs), which may be involved in the development of nonalcoholic fatty liver disease (NALFD) and drug-induced liver injury (DILI). In this study, we investigated the presence of *C. difficile*-derived MVs in patients with and without CDI, and analyzed their effects on pathways related to NAFLD and DILI in HepG2 cells. Fecal extracellular vesicles from CDI patients showed an increase of *Clostridioides* MVs. *C. difficile*-derived MVs that were internalized by HepG2 cells. Toxigenic *C. difficile*-derived MVs decreased mitochondrial membrane potential and increased intracellular ROS compared to non-toxigenic *C. difficile*-derived MVs. In addition, toxigenic *C. difficile*-derived MVs upregulated the expression of genes related to mitochondrial fission (FIS1 and DRP1), antioxidant status (GPX1), apoptosis (CASP3), glycolysis (HK2, PDK1, LDHA and PKM2) and β-oxidation (CPT1A), as well as anti- and pro-inflammatory genes (IL-6 and IL-10). However, non-toxigenic *C. difficile*-derived MVs did not produce changes in the expression of these genes, except for CPT1A, which was also increased. In conclusion, the metabolic and mitochondrial changes produced by MVs obtained from toxigenic *C. difficile* present in CDI feces are common pathophysiological features observed in the NAFLD spectrum and DILI.

## 1. Introduction

Changes in the gut microbiota are associated with the development and pathogenesis of different liver diseases [1,2,3]. Gut bacteria play a key role in metabolic and immune health. When the integrity of the intestinal barrier is disrupted, some microbial communities and/or their metabolites can easily reach the liver, causing high-level toxin exposure and leading to hepatocyte injury. In the liver, bacteria or their metabolites can stimulate hepatic immune cells and activate inflammatory pathways, oxidative stress and mitochondrial dysfunctions [4,5].

Antimicrobial drug treatment often induces dysbiosis in microbial communities, resulting in increased susceptibility to colonization by opportunistic intestinal pathogens [4]. This dysbiosis may promote the disruption of the integrity of the intestinal barrier. *Clostridioides difficile* (*C. difficile*) is recognized as the leading cause of bacterial nosocomial diarrhea, strongly associated with prior antibiotic use [6]. Like other Gram-positive bacteria, *C. difficile* is capable of secreting extracellular vesicles (membrane vesicles (MVs) of Gram-positive bacteria) [7] of a size considerably smaller than the size of the bacteria. These MVs are lipidic nanovesicles (~25–300 nm) derived from bacterial cells and can contain various molecules, such as nucleic acids (DNA, RNA), proteins, lipids or organic molecules, among others [8,9]. The role of microbiota-derived EVs is receiving much attention, due to their immunomodulatory and regulatory capacity for different cell signaling pathways [10,11]. Furthermore, given the stability and distribution of EVs, they have also been proposed as indicators of the host microbiota [12]. The microbial composition determined by MVs metagenomics could be a reflection of gut microbes that can produce MVs containing DNA within them. In some cases, the metagenomic composition of these MVs is similar to that of the source feces, but in other cases there may be some differences, mainly in certain diseases such as obesity and Crohn’s disease [13]. These EVs can interact in the gut by delivering components such as bacterial small RNA and proteins to host cells, which differ in abundance and composition depending on pathological conditions [14]. *C. difficile*-derived MVs are able to stimulate the expression of pro-inflammatory cytokine genes and induce cytotoxicity in human Caco-2 colorectal epithelial cells [8]. The effects of these *C. difficile*-derived MVs may contribute, together with TcdA and TcdB toxins, to intestinal mucosal injury. In addition, *C. difficile*-derived MVs have also been shown to induce the release of inflammatory cytokines. However, how bacterial EVs interact with human cells at some anatomical locations has been scarcely studied. So-called hypervirulent strains of *C. difficile*, such as NAP1/027, produce the actin-ADP ribosylating toxin known as CDT or binary toxin. CDT causes the formation of long-microtube-like protrusions and increases host cell adhesion [15]. A recent study shows that these protrusions contain membrane vesicles, suggesting vesicular trafficking and secretory processes associated with the presence of this toxin [16]. Studies have described the selective export of toxins and other virulence factors and their transport to host cells via EVs. These EVs may protect toxins and virulence factors from host defenses and allow them to reach host cells more efficiently [17]. It has also been reported that the secretion of EVs can modify mitochondrial functions through the uptake of their contents, promoting inflammation, cancer progression or the recovery of cellular function from damaged mitochondria [18].

*C. difficile* infection (CDI) is the main cause of healthcare-associated diarrhea, while nonalcoholic fatty liver disease (NAFLD) is the most common cause of chronic liver disease. However, the relationship between these two entities is not entirely clear. *C. difficile* infection (CDI) has been frequently reported in patients with liver disease, such as NALFD and cirrhosis [19,20]. In addition, a previous study demonstrated that *C. difficile* overgrowth in the gut promotes liver inflammation in mice [21]. Since the bacterial translocation of *C. difficile* was not detected in all liver samples studied, other possible pathogenic pathways, such as MVs, should be investigated. However, to date there have been no studies analyzing the possible effect of *C. difficile*-derived MVs on liver cells. These MVs could be modifying metabolic pathways involved in the development of different liver pathologies, such as NALFD and drug-induced liver injury (DILI). These diseases seem to share molecular mechanisms related to mitochondria, which are involved in numerous functions including energy metabolism, lipid synthesis, and autophagy regulation [22]. Recently, several bacterial pathogens have been shown to modulate mitochondrial function [23].

Therefore, the aim of this study was, firstly, to explore the composition of fecal microbiota-derived EVs in patients with and without CDI, to detect the possible presence of *C. difficile*-derived MVs. Secondly, we investigated in vitro the relationship of *C. difficile*-derived MVs with the development of alterations in HepG2 cells related to the development of different liver diseases.

## 2. Materials and Methods

### 2.1. Patients’ Recruitment

The cohort study included 55 patients: 28 patients with diarrhea due to causes other than CDI, and 27 patients with diarrhea related to CDI. Patient samples were processed and frozen at −80 °C immediately after receipt at the Virgen de la Victoria University Hospital (Málaga, Spain). All participants were of Caucasian origin. All participants gave written informed consent, the study protocol was conducted in accordance with the ethical guidelines of the Declaration of Helsinki and the study was approved by the Malaga Provincial Research Ethics Committee, Malaga, Spain (UMA18-FEDERJA-194 and PI18/01652).

### 2.2. Isolation of EVs from Human Feces

A total of 10 g of feces was inoculated into 40 mL of sterile phosphate-buffered saline (PBS) and homogenized. Next, EVs were then isolated by centrifugation as previously described, with some modifications [8,13,24]. A first centrifugation of the homogenate was performed (40 min, 4000× *g*, 4 °C), and the supernatant was recovered and filtered using sterilized vacuum filtration units, Rapid-Flow™ filters MF 75, and Nalgene^®^ 0.2 μm in cold ice (ThermoFisher Scientific, Waltham, MA, USA). The filtrate was transferred to 10 mL polycarbonate open top thick wall tubes and ultracentrifuged at 100,000× *g* for 3 h at 4 °C, with a fixed angle rotor Type 70.1 Ti in a Beckman Optima XL-100K ultracentrifuge (Beckman Coulter Life Sciences, Lakeview Pkwy S Drive, Indianapolis, IN, USA). Pellets were resuspended in 200 µL of PBS and the EVs were purified using qEVoriginal size exclusion columns (SEC) of 70 nm (Izon Science Europe Ltd., Oxford, UK) [25,26], following the manufacturer’s recommendations. Fractions 7–10 were collected, mixed, concentrated with Vivaspin^®^ 20 100 kDa (Sartorius Stedim Biotech GmbH, Göttingen, Germany) centrifugal concentrators, aliquoted and frozen at −80 °C until used.

### 2.3. DNA Extraction, Libraries Preparation and Sequencing

Total DNA was extracted from EVs using DNeasy blood and Tissue Kits (QIAGEN Science, Hilden, Germany), following the manufacturer´s recommendations. The DNA was eluted into DNase/RNase-free water and its concentration and purity were evaluated using a NanoDrop ND-1000 spectrophotometer (NanoDrop Technologies, Inc., Wilmington, DE, USA). Extracts were stored at −20 °C until used. Libraries and sequencing were performed as previously described [27]. Briefly, the amplification of V1-V3 regions of the 16S rRNA bacterial gene was performed using the primers 5′-GAGAGTTTGATYMTGGCTCAG-3′ forward and 5′-ACCGCGGCTGCTGGCAC-3′reverse with overhand adapters. Amplicons were purified using Agencourt AMPure XP bead kit (Beckman Coulter, Pasadena, CA, USA), indexed using Nextera XT index primers 1 and 2 (Illumina, San Diego, CA, USA), quantified by Quant-IT PicoGreen (Thermo Fisher Scientific, Waltham, MA, USA) and diluted to a concentration of 10 ng/μL. DNA samples were quantified by qPCR with KAPA 170 SYBR^®^ FAST qPCR Kit (Kapa Biosystems, Wilmington, MA, USA). Samples were normalized, pooled and sequenced using Illumina MiSeq technology with v3 reagents (Illumina, San Diego, CA, USA), using paired end reads, with the GIGA Genomics platform (Liège, Belgium). Negative controls were used in the entirety for DNA extraction, library preparation and sequencing.

### 2.4. Bioinformatics, Ordination and Statistical Analysis for the Metagenomic Study

Sequence reads were processed using, respectively, Mothur v1.44.3 and VSearch for alignment, clustering and chimera detection [28,29]. Sequences were clustered into operational taxonomic units (OTUs) at 97% of identity. The SILVA 138 database of full-length 16S rDNA gene sequences was used for alignments of unique sequences and taxonomical assignations. Finally, cleaned sequences were rarefied to 10,000 reads per sample. Original libraries are publicly available under the BioProject PRJNA945623 at the National Center for Biotechnology Information (NCBI) (http://www.ncbi.nlm.nih.gov/bioproject/945623 (accessed on 17 March 2023)).

Analyses were performed at phylum, family and genus level. Regarding alpha diversity (reciprocal Simpson diversity index and Simpson evenness), Goods’s coverage and population richness (Chao1 estimator of richness) were calculated using Mothur v1.44.3 and compared between two groups using a Mann–Whitney test (PRISM 8). Barplots were built using PRISM 8, including only genera with relative abundance >1%. The bacterial community structure was visualized with non-parametric dimensional scaling (k = 4, model stress = 0.089) based upon the Bray–Curtis dissimilarity matrix using vegan and vegan3d packages in RStudio (2022.12.0+353). Post hoc pairwise differences between groups were assessed with Deseq2 package in R using Benjamini–Hochberg FDR correction [30].

### 2.5. Cell and Culture Conditions

The HepG2 cell line was obtained from human hepatocarcinoma (ATCC^®^HB-8065™, 85011430-1VL, Merck LifeScience S.L.U., Darmstadt, Germany) and cultured in low-glucose Dulbecco’s modified Eagle’s medium (DMEM) (Gibco Carlsbad, CA, USA) supplemented with 10% inactivated fetal bovine serum (FBS) (Gibco Carlsbad, CA, USA), 1% penicillin/streptomicine (Merck LifeScience S.L.U., Darmstadt, Germany) and 2 mM glutamine (Gibco, Carlsbad, CA, USA). Cells were cultured in a humidified atmosphere at 37 °C, 21% O_2_ and 5% CO_2_. Cells were sub-cultured by dispersion using a trypsin/EDTA solution (Gibco, Carlsbad, CA, USA) when they reached about 80% confluence every 72 h.

### 2.6. C. difficile Culture and MVs Production

The *C. difficile* strains used in this study were isolated from human feces and belonged to PCR ribotypes 078 (toxigenic type) and 039 (non-toxigenic type), following the Cardiff ribotype nomenclature (Anaerobic Reference Unit (ARU), UK) from the strain collection available at the Catholic University of Leuven (National Reference Laboratory for *C. difficile* in Belgium). Strains were characterized for the presence of A, B and CDT toxin genes by multiplex PCR, as previously described [31]. These PCR ribotypes were subsequently analyzed using the WEBRIBO web database [32]. Strains were preserved frozen (−80 °C) in a 20% glycerol 80% Brain Heart Infusion Broth solution (Oxoid Limited, Hampshire, UK).

For MVs production, 1 mL aliquot was placed in 40 mL of regenerated BHI broth without antibiotics and incubated at 37 °C for 7 days under strict anaerobic conditions. The anaerobic atmosphere in the flask was created using AnaeroGen™ sachet (Oxoid Limited, Hampshire, UK) and checked using an anaerobic indicator BR0055B (Oxoid Limited, Hampshire, UK). MVs were then isolated by centrifugation as described for human feces [8,23], with a slight modification. An aliquot of MVs was purified using qEVoriginal size exclusion columns of 70 nm (Izon Science Europe Ltd., Oxford, UK) [25,26], following the manufacturer´s recommendations, to perform an optimal separation of MVs from most of the other contaminating soluble molecules, such as toxins and proteins [25,26] (purified MVs). Fractions 7–10 were collected, mixed, concentrated with Vivaspin^®^ 20 100 kDa (Sartorius Stedim Biotech GmbH, Göttingen, Germany) centrifugal concentrators, aliquoted and frozen at −80 °C until used. A second aliquot was used without this purification method (non-purified MVs). The protein concentration of MVs was determined using the bicinchoninic acid (BCA) assay (Thermo Fisher Scientific, Waltham, MA, USA). The presence of GDH (Glutamate Dehydrogenase Enzyme) and toxin B was assessed using a chromatographic immunoassay (CerTest, Biotec, Spain), following the manufacturer´s instructions. The confirmation of the presence of *C. difficile* DNA was performed by detection of a species-specific internal fragment of the *tpi* gene by classical polymerase chain reaction (PCR), as described in a previous study [33]. All PCR reactions were run with positive and negative controls.

### 2.7. Electron Microscopy of EVs

Isolated MVs were fixed in 2% paraformaldehyde—0.1 M PBS for 30 min. The glow discharge technique (60 s, 7.2 V, using a Bal-Tec MED 020 Coating System) was applied over carbon-coated copper grids, and these grids were immediately placed on top of sample drops for 15 min. Then, the grids with adherent MVs were washed in a 0.1 M PBS drop and additional fixation in 1% glutaraldehyde was performed for 5 min. After washing properly in distilled water, the grids were contrasted with 1% uranyl acetate and embedded in methylcellulose. Excess fluid was removed and allowed to dry before examination with a transmission electron microscope FEI Tecnai G2 Spirit (ThermoFisher Scientific, Waltham, MA, USA). All images were obtained using a Morada digital camera (Olympus Soft Image Solutions GmbH, Münster, Germany).

### 2.8. Cellular Exposition to C. difficile-Derived MVs

When HepG2 cells reached approximately 80% confluence, they were exposed to 15 and 30 µg of protein/mL [34] of *C. difficile*-derived MVs for 24 h, after which different experiments were performed, as described below. To confirm that part of the effects of non-purified and toxigenic *C. difficile*-derived MVs may be due to TcdB, we incubated HepG2 cells with 30 µg of protein/mL of purified and toxigenic *C. difficile*-derived MVs and 3 ng/mL of TcdB (Sigma-Aldrich, St. Louis, MO, USA) for 3 h, a dose according to a previous study [35].

### 2.9. Fluorescence Microscopy Study of C. difficile-Derived MVs Internalization

*C. difficile*-derived MVs (30 µg of protein/mL) were stained with PKH26 Red Fluorescent Cell Linker Kits (PKH26GL, Sigma-Aldrich, St. Louis, MO, USA) according to the manufacturer’s instructions. Briefly, MVs were suspended with Diluent C, mixed with 1ml of PKH26 solution (4 μL of red dye in 1ml of diluent C) and incubated for 5 min while mixing with gentle pipetting. Staining was then stopped by adding an equal volume of 10% exosome-depleted fetal bovine serum (Sigma-Aldrich, St. Louis, MO, USA) in Dulbecco’s modified Eagle’s medium (Sigma-Aldrich, St. Louis, MO, USA) and incubating for 1 min. They were then diluted to 2 mL with phosphate-buffered saline (PBS), transferred to Vivaspin^®^ 20, 100 kDa (Sartorius Stedim Biotech GmbH, Göttingen, Germany), and centrifuged at 4000× *g* for 3 min at 4 °C. MVs were washed three times with 2 mL PBS before being concentrated to a final volume of 50 μL. Alternatively, particle-free PBS (Sigma-Aldrich, St. Louis, MO, USA) was used instead of *C. difficile*-derived MVs to obtain a PKH26-labeled MVs-free control, which was used as a negative control for fluorescence microscopy.

The uptake of *C. difficile*-derived MVs by HepG2 cells was assessed on Chambered Coverslips (Sigma-Aldrich, St. Louis, MO, USA) by fluorescence microscopy. After incubation of HepG2 cells with PKH26-labeled *C. difficile*-derived MVs for 24 h, the cells were fixed with 4% paraformaldehyde solution for 20 min at room temperature with gentle agitation, and washed twice with PBS. In parallel to MVs labelling, we stained lysosomes using a Lysosomal Staining Kit-Green Fluorescence-Cytopainter (ab112136, Abcam, Cambridge, UK) according to the manufacturer’s instructions. Cell nuclei were counterstained with UltraCruz™ Mounting Medium for fluorescence with DAPI (Santa Cruz Biotechnology, Santa Cruz, CA, USA) and analyzed with an Olympus BX51 microscope equipped with an Olympus DP70 digital camera (Olympus, Glostrup, Denmark). Immunohistochemical techniques were performed in the Imaging platform of the Institute of Biomedical Research in Malaga, Spain (IBIMA).

### 2.10. Mitochondrial Membrane Potential Assessment

Mitochondrial membrane potential (MMP) was analyzed using the tetramethylrhodamine methyl ester (TMRM) dye in live cells (TMRM Assay Kit (Mitochondrial Membrane Potential) ab228569, Abcam, Cambridge, UK). Briefly, after incubation with MVs in a 96 wells microplate, HepG2 cells (2 × 10^5^/mL) were incubated with 500 nM TMRM at 37 °C for 45 min. Cells were first washed with 100 μL of PBS/0.2% BSA, and then washed with 1X Live Cell Imaging Buffer. Fluorescence measurement was obtained using a Microplate Fluorescence Reader (BioTek Instruments, Inc., model: FL600 FA, Winooski, VT, USA) at excitation and emission of 548 and 573 nm, respectively. Carbonyl cyanide p-(tri-fluromethoxy) phenyl-hydrazone (FCCP), a potent uncoupler of oxidative phosphorylation in mitochondria, was used as a positive control. Fluorescence was presented as TMRM fluorescence intensity with arbitrary units. Data were normalized using DAPI fluorescence intensity.

### 2.11. Intracellular ROS Level Measurement

Intracellular ROS production was measured using dihydroethidine (DHE) dye (Assay Kit Reactive Oxygen Species (ab236206), Abcam, Cambridge, UK) in live cells, which is selective for superoxide anion and hydrogen peroxide. Briefly, following incubation with MVs in a 96 wells microplate, HepG2 cells (2 × 10^5^/mL) were incubated with DHE (2.5 μM) for 30 min at 37 °C. Cells were washed, resuspended in PBS and analyzed for fluorescence intensity using a Microplate Fluorescence Reader (BioTek Instruments, Inc., model: FL600 FA, Winooski, VT, USA) at excitation and emission wavelengths of 480–520 nm and 570 nm, respectively. Antimycin A, an inhibitor of complex III of the mitochondrial electron transport chain, was included as a positive control for ROS generation. N-acetyl Cysteine was included as an anti-oxidant control. The level of ROS generation was represented as total DHE fluorescence intensity. Data were normalized using DAPI fluorescence intensity.

### 2.12. Quantification of Adherent Cells

To normalize the results of intracellular ROS level and TMRM signal, the number of cells after each MVs treatment was quantified. For adherent cells in 96-well cell culture plates, we first washed the monolayers twice with 1x PBS. Then, cell nuclei were counterstained with UltraCruz™ Mounting Medium for fluorescence with DAPI (Santa Cruz Biotechnology, Santa Cruz, CA, USA) for microscopy and DAPI (1–1000) for microplate analyzed with a fluorescence plate reader (BioTek Instruments, Inc., model: FL600 FA, Winooski, VT, USA), using 350 nm excitation and 486 nm emission wavelengths. The instructions described in the method related to the quantification of fixed adherent cells using the fluorescence of DNA dyes were followed [36].

### 2.13. RNA Isolation and Quantitative Real-Time PCR

Total RNA from HepG2 cells was extracted with an RNeasy mini kit (QIAGEN Science, Hilden, Germany). First strand cDNA was synthesized by retrotranscription using M-MLV retrotranscriptase (Promega, Madison, WI, USA). Gene expression levels were analyzed in triplicate by real-time PCR using a SensiFAST SYBR Green Kit (Bioline, London, UK) in a 7500 Fast (Thermo Fisher Scientific Inc. Waltham, MA, USA). Primers for the PCR reaction were designed using the primer design tool from IDT (Integrated DNA Technologies, Coralville, IA, USA). The primers used in the current study are described in Table 1. The relative expression levels of all genes were normalized to the expression of the house-keeping gene (β-actin), and data were analyzed with the ∆∆C_T_ method.

### 2.14. Statistical Analyses

The statistical analysis for the metagenomic study has been described above. For the other statistical analyses performed in the study, data were analyzed with GraphPad Software (Prism 8.1.1) (GraphPad Software, San Diego, CA, USA). Differences between groups were compared using Kruskal–Wallis tests followed by post hoc analyses using the Dunn’s test. Values were considered statistically significant when *p* < 0.05.

## 3. Results

### 3.1. Microbiome Profile of EVs from Patients with and without CDI

**Figure 1:**
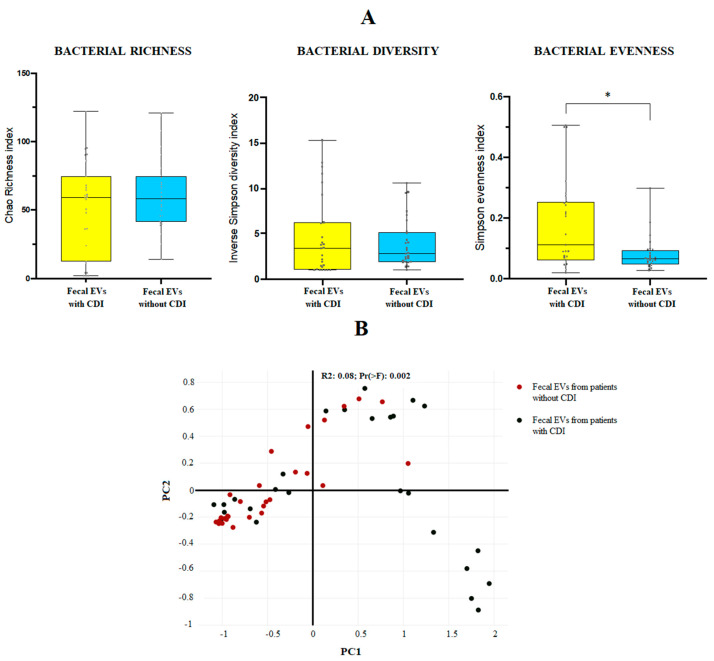
(**A**) Bacterial diversity (Inverse Simpson diversity index), bacterial evenness (Simpson evenness index) and bacterial richness (Chao1 richness index) expressed as the mean value with standard deviation for fecal EVs isolated from human feces of patients with and without *C. difficile* infection (CDI). * *p* < 0.05. Statistical differences are calculated according to Mann–Whitney test. (**B**) Sample distribution in function of the sample group using three-dimensional dynamic ordination. Beta-diversity analysis using a Bray–Curtis-based NMDS model of beta-diversity. Red circle: fecal EVs samples obtained from patients without CDI; black circle: fecal EVs samples obtained from patients with CDI.

The 16S amplicon sequencing yielded 10.000 identifications per sample. For the mean bacterial richness (Chao 1 richness index) and alpha bacterial diversity (Inverse Simpson diversity index), no significant differences were found between the two groups at genus level. Regarding the population evenness (Simpson evenness index), significant differences (*p*-value 0.0095) were detected between CDI and non-CDI EVs, showing an increase in CDI samples (Figure 1A). The Bray–Curtis dissimilarity matrix is shown in Figure 1B.

**Figure 2:**
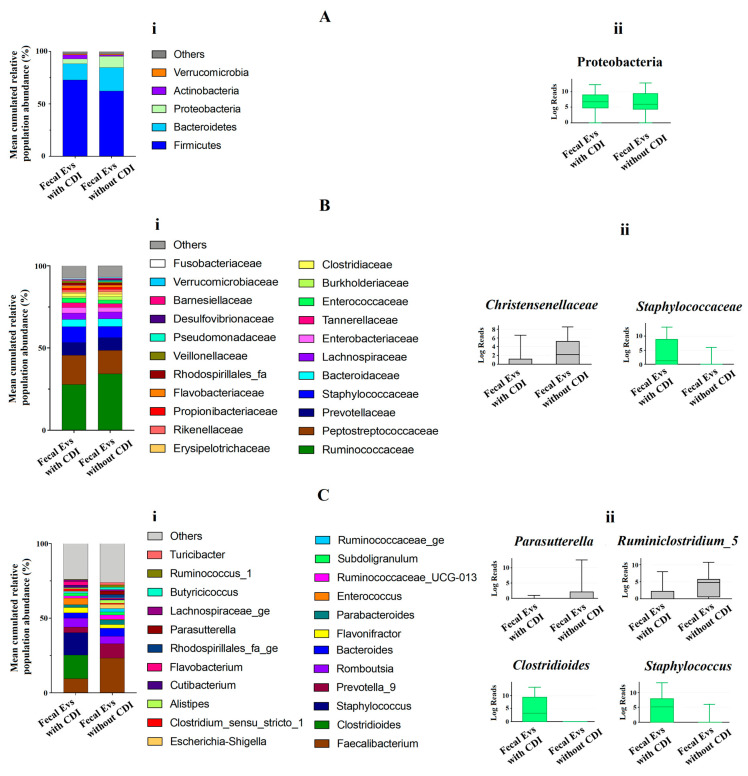
(**Ai**,**Bi**,**Ci**) Metagenomics profiles of fecal EVs isolated from patients with and without *C. difficile* infection (CDI). (**Ai**) Composition at phylum level with bar chart detailing the mean cumulative relative abundance (%) of the top 5 phyla common to the two patient groups. (**Bi**) Composition at family level with bar chart detailing the mean cumulative relative abundance (%) of the top 22 bacterial families common to the two patient groups. (**Ci**) Composition at genus level with bar chart detailing the mean cumulative relative abundance (%) of the 23 core genera common to the two groups of patients. Only phyla, families and genera with relative abundance >1% were plotted. (**Aii**,**Bii**,**Cii**) Box plots showing bacterial phyla (**Aii**), families (**Bii**) and genus (**Cii**) whose relative abundances were >1% and with statistical differences between fecal EVs isolated from patients with CDI and without CDI. Results of DESeq2 with Benjamini–Hochberg FDR corrections. Log Reads: Log2 relative normalized abundance. *Staphylococcaceae, Staphylococcus* and *Clostridioides* appear in green since they are a part of the 10 genera with the highest relative abundance in the analyzed samples.

At phylum level, the metagenomic composition of EVs showed a predominance of Firmicutes, Bacteroidetes, Proteobacteria and Actinobacteria in both types of samples studied (Figure 2Ai). Significant differences were only detected for Proteobacteria, with an increase of this phylum in EVs samples from non-CDI patients compared to CDI patients (Figure 2Aii). Figure 2Bi illustrates the metagenomic composition at family level. EVs samples from CDI patients showed a statistically significant increase of the *Staphylococcaceae* family, but a decrease in the *Christensenellaceae* family (Figure 2Bii). At genus level, twenty-three genera had a relative abundance exceeding 1% in both types of samples (Figure 2Ci). A significant increase of *Clostridioides* and *Staphylococcus,* and a decrease of *Parasutterella* and *Ruminiclostridium*, were detected in EVs samples from patients with CDI (Figure 2Cii).

### 3.2. EVs from C. difficile Are Internalized by HepG2 Cells

**Figure 3:**
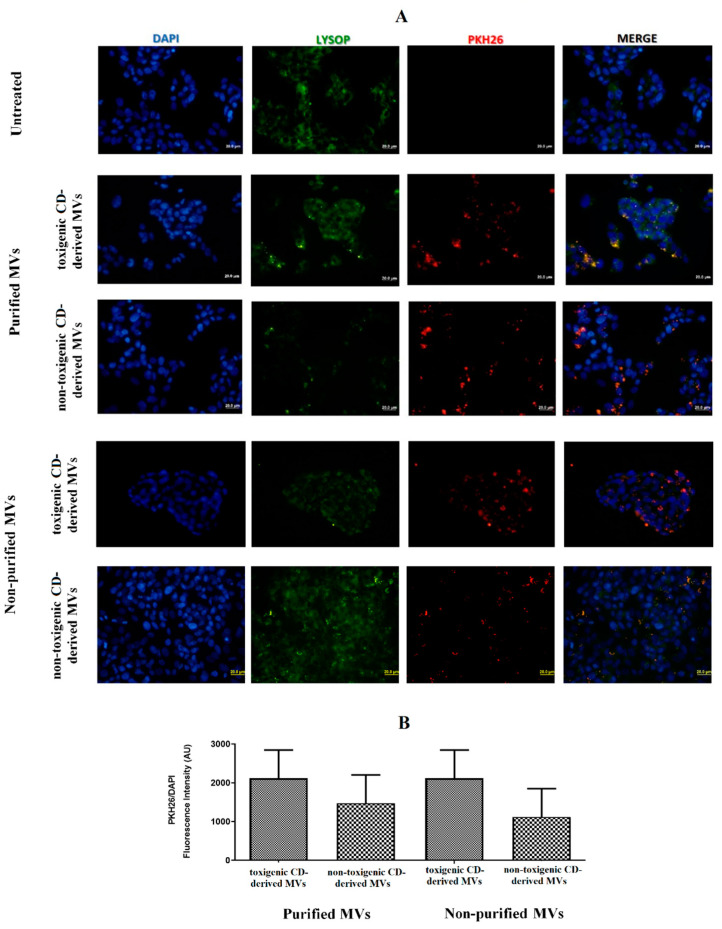
MVs from *C. difficile* (CD) are taken up by HepG2 cells. (**A**) HepG2 cells were incubated with PKH26-labeled MVs (30 µg total protein/mL) for 24 h. MVs from non-toxigenic and toxigenic *C. difficile* strains, both non-purified and purified by SEC, were used. After incubation, nuclear DNA was stained with DAPI, and lysosomes were stained with Lysosomal Staining Kit-Green Fluorescence-Cytopainter. Cells were visualized with an Olympus BX51 (20×). (**B**) PKH26/DAPI ratio fluorescence intensity at the different conditions used in the incubations with *C. difficile*-derived MVs. Data represent mean ± SEM (*n* = 3).

Once the presence of *C. difficile*-derived MVs was detected in human feces, we wanted to assess the effects of these *C. difficile*-derived MVs on HepG2 cells. First, we studied whether PKH26-labeled MVs are uptake up for HepG2 cells. For this study, we carried out two different methodologies to isolate MVs from *C. difficile*, as mentioned in the Materials and Methods section (non-purified and purified MVs based on the use of SEC). After 24 h of exposure, we found an intracellular localization of purified and non-purified MVs close to the cellular nucleus (Figure 3A). Moreover, we assessed a slightly higher number, although not significant, of toxigenic *C. difficile*-derived MVs with intracellular localization, as measured by the PKH26/DAPI ratio fluorescence intensity, compared with non-toxigenic *C. difficile*-derived MVs (Figure 3B).

### 3.3. Effects of Non-Purified C. difficile-Derived MVs on the Morphology of HepG2 Cells

**Figure 4:**
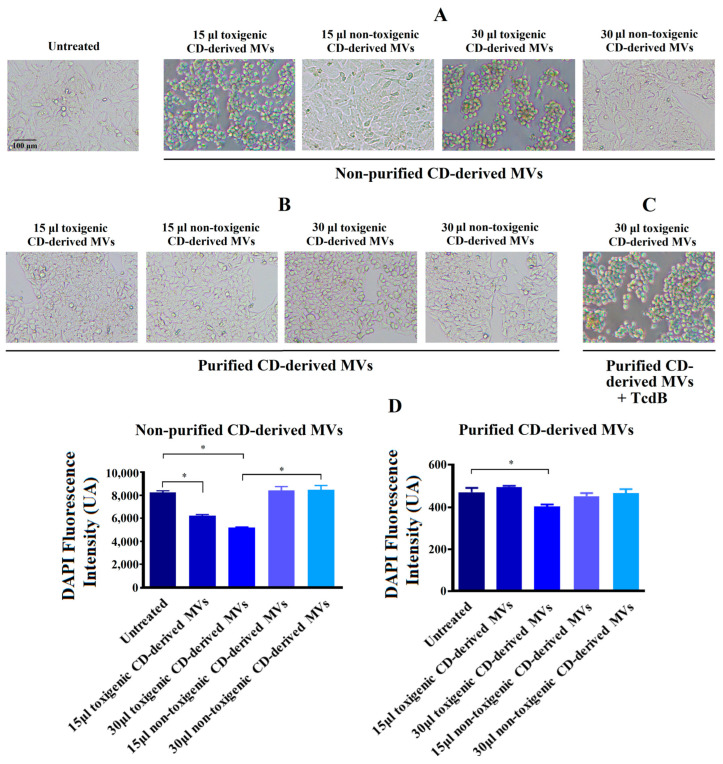
Changes produced by *C. difficile* (CD)-derived MVs on the HepG2 cells’ morphology and quantification of fixed adherent cells by the DAPI staining. HepG2 cells were treated with 15 and 30 μg/mL of non-toxigenic and toxigenic *C. difficile*-derived MVs during 24 h. (**A**) Effect of non-purified *C. difficile*-derived MVs. (**B**) Effect of purified *C. difficile*-derived MVs. (**C**) HepG2 cells treated with purified and toxigenic *C. difficile*-derived MVs and TcdB (3 ng/mL). (**D**) Quantification of adherent cells measured by changes in DAPI fluorescence intensity in HepG2 cells after MVs incubation. Data represent mean ± SEM (*n* = 3). * *p* < 0.05.

In order to explore the effects of *C. difficile*-derived MVs on HepG2 cells, we first analyzed the morphology of these cells after 24 h of exposure. In Figure 4A, we observed a significant change in cellular morphology in HepG2 cells treated during 24 h with 15 and 30 μg/mL of non-purified toxigenic *C. difficile*-derived MVs. After this incubation, a rounded and smaller shape was observed. In addition, we observed a significant decrease in the DAPI fluorescence intensity with 15 and 30 μg/mL of non-purified toxigenic *C. difficile*-derived MVs, which was related to the cytotoxic effects and cell death rate [36,37] (Figure 4C). Meanwhile, non-purified and non-toxigenic *C. difficile*-derived MVs maintained a morphology similar to that of untreated control cells.

### 3.4. Effects of Purified C. difficile-Derived MVs on the Morphology of HepG2 Cells

**Figure 5:**
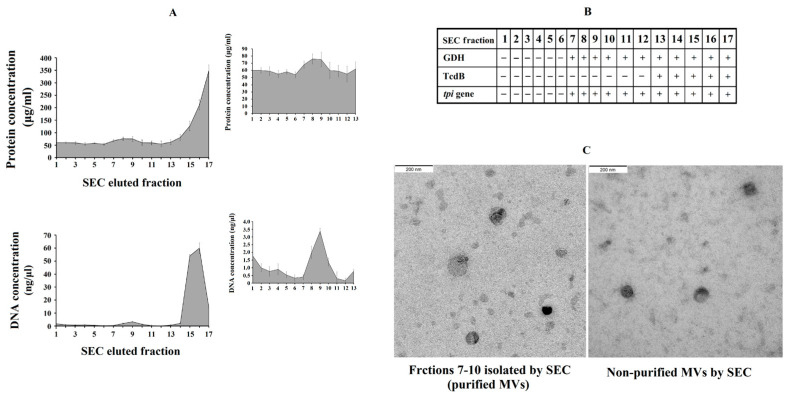
Analysis of *C. difficile*-derived MVs-rich eluted fractions after size exclusion chromatography (SEC). (**A**) Protein and total DNA concentrations of SEC eluted fractions with the magnified part of *C. difficile*-derived MVs-rich fractions in the upper-right corner. (**B**) Scheme showing the presence (+) or absence (-) of Glutamate Dehydrogenase Enzyme (GDH), toxin B (TcdB) and *tpi* gene in the SEC eluted fractions. (**C**) Characterization by electronic microscopy of the non-purified and purified (fractions 7–10) *C. difficile*-derived MVs. Data represent mean ± SEM (*n* = 3).

We further explored the effect of purified *C. difficile*-derived MVs. We hypothesized that the methodology used to obtain non-purified MVs played a role in the effect described above. Thus, the presence of Toxin B in non-purified *C. difficile*-derived MVs was revealed using rapid chromatographic immunoassay. Meanwhile, the purification of MVs by SEC removed most of the remaining components that were collected when MVs were isolated (proteins and TcdB toxin) (Figure 5A), and other components of the culture supernatant which have been shown to induce inflammatory responses [25,26]. We collected those elution fractions that contained GDH and DNA from *C. difficile* (*tpi* gene) (Figure 5B), but they did not contain TcdB toxin (Figure 5B), and contained more total DNA (Figure 5A), proteins (Figure 5A) and MVs observed by electron microscopy (Figure 5C) (fractions 7–10).

The exposure of HepG2 cells to 30 μg/mL of purified and toxigenic *C. difficile*-derived MVs for 24 h only induced a slight change in cell morphology (Figure 4B). To confirm that the different cell morphology found between HepG2 cells treated with non-purified and purified, both toxigenic, *C. difficile*-derived MVs was due to TcdB, we treated HepG2 cells with purified and toxigenic *C. difficile*-derived MVs and TcdB, resulting in a rounding of HepG2 cells (Figure 4C). In addition, we detected a significant decrease in DAPI fluorescence intensity after exposure to 30 μg/mL of purified and toxigenic *C. difficile*-derived MVs (Figure 4D). Meanwhile, the HepG2 cells treated with purified and non-toxigenic *C. difficile*-derived MVs maintained a similar morphology to untreated control cells (Figure 4A,B) and did not decrease the DAPI fluorescence intensity (Figure 4D).

### 3.5. C. difficile-Derived MVs Induce Mitochondrial Dysfunction and Increase ROS Level in HepG2 Cells

Due to the significant undesirable effects produced by non-purified *C. difficile*-derived MVs on cell morphology and the DAPI fluorescence detected in HepG2 cells, we sought to explore the effects of non-purified and purified *C. difficile*-derived MVs on mitochondrial function, through the assessment of MMP, and ROS production.

**Figure 6:**
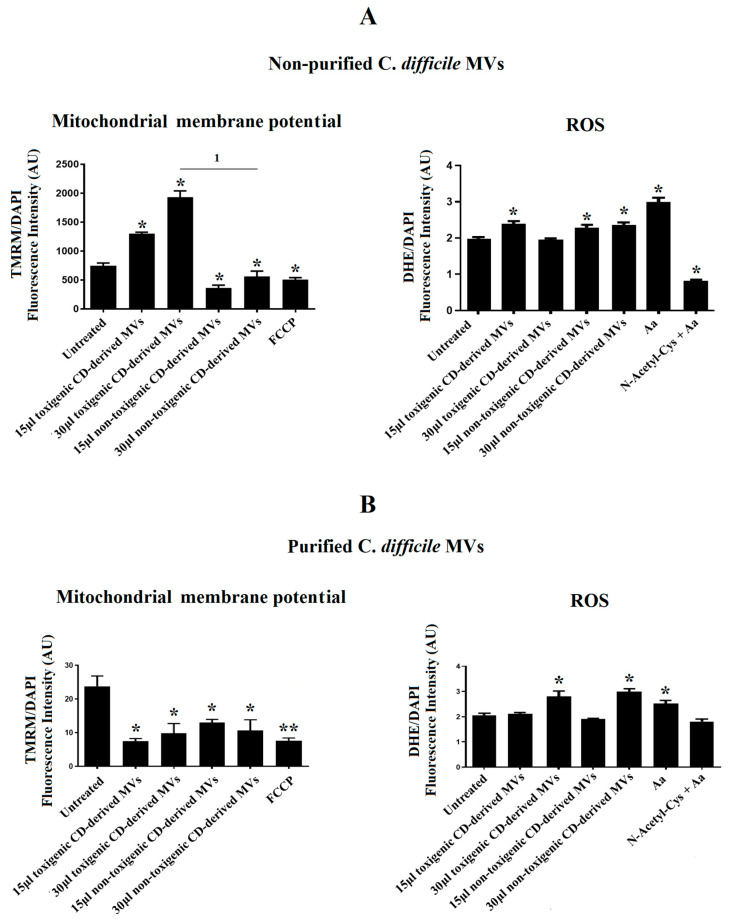
Effects of *C. difficile*-derived MVs (30 µg total protein/mL) on the mitochondrial membrane potential (MMP) and intracellular ROS levels in HepG2. TMRM staining was used to determine the MMP in HepG2 cells. DHE staining was used to assess intracellular ROS level in live cells. (**A**) Effects of non-purified *C. difficile*-derived MVs. (**B**) Effects of purified *C. difficile*-derived MVs. * *p* < 0.05 and ** *p* < 0.001 as compared with untreated cells (*n* = 6). ^1^ *p* < 0.05. FCCP: Carbonyl cyanide *p*-(tri-fluromethoxy) phenyl-hydrazone. aa: Antimycin A.

After 24 h of co-culture with 15 and 30 μg/mL of non-purified *C. difficile*-derived MVs, we observed an increase in MMP with 15 and 30 μg/mL of toxigenic *C. difficile*-derived MVs (*p* = 0.042 and *p* = 0.024, respectively), while non-toxigenic *C. difficile*-derived MVs decreased MMP (*p* = 0.041 and *p* = 0.045, respectively) (Figure 6A). In addition, we detected an increase in intracellular ROS generation, by quantification of DHE fluorescent signal, in HepG2 cells treated with 15 μg/mL of toxigenic *C. difficile*-derived MVs (*p* = 0.043) and with 15 and 30 μg/mL of non-toxigenic *C. difficile*-derived MVs (*p* = 0.047 and *p* = 0.045, respectively) (Figure 6A).

After 24 h of co-culture with purified *C. difficile*-derived MVs, we observed a decrease in MMP with 15 and 30 μg/mL of toxigenic *C. difficile*-derived MVs (*p* = 0.027 and 48, respectively) and with 15 and 30 μg/mL of non-toxigenic *C. difficile*-derived MVs as compared with untreated cells (*p* = 0.034 and *p* = 0.047) (Figure 6B). In addition, we detected an increase in intracellular ROS generation in cells treated with 30 μg/mL of toxigenic and non-toxigenic *C. difficile*-derived MVs (*p* = 0.048 and *p* = 0.037, respectively) compared with untreated cells (Figure 6B).

### 3.6. Mitochondrial Fission Key Genes Are Up-Regulated by Purified Toxigenic C. difficile-Derived MVs in HepG2 Cells

**Figure 7:**
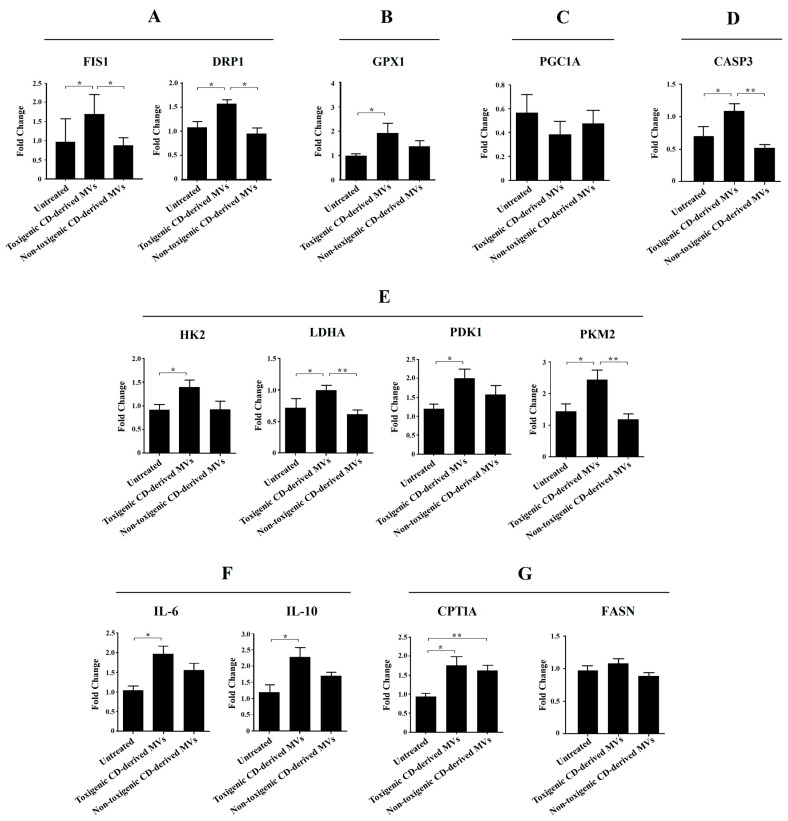
Effects of purified *C. difficile*-derived MVs (30 µg total protein/mL) on the expression of genes related to (**A**) mitochondrial fission (FIS1 and DRP1), (**B**) antioxidant state (GPX1), (**C**) mitochondrial biogenesis (PGC1A), (**D**) apoptosis (CASP3), (**E**) glycolysis (HK2, PKM2, LDHA and PDK1), (**F**) inflammation (IL-6 and IL-10), and (**G**) β-oxidation (CPT1A) and synthesis of fatty acids (FASN) in HepG2 cells. The plot shows the mean of the relative expression ± SEM (*n* = 6). * *p* < 0.05, ** *p* < 0.01. DRP1: dynamin-related protein. FIS1: mitochondrial fission 1 fission. GPX1: glutathione peroxidase 1. LDHA: lactate dehydrogenase A. HK2: hexokinase 2. PDK1: pyruvate dehydrogenase kinase 1. PKM2: pyruvate kinase M2. PGC1A: peroxisome proliferator-activated receptor γ co-activator 1 α. CASP3: caspase 3. CPT1A: carnitine palmitoyltransferase 1A. FASN: fatty acid synthase. IL-6: interleukin-6. IL-10: interleukin-10.

Due to the significant undesirable effects induced by non-purified *C. difficile*-derived MVs on HepG2 cell morphology and DAPI fluorescence, here we focused on exploring the effects of purified *C. difficile*-derived MVs on liver mitochondria, and with the dose that produced the most significant results (30 μg/mL of *C. difficile*-derived MVs). We evaluated the expression of mitochondrial dynamics (mitochondrial fission: FIS1 and DRP1) and antioxidant (GPX1) genes in HepG2 cells (Figure 7).

We found a significant increase in FIS1, DRP1 and GPX1 mRNA in HepG2 cells exposed to toxigenic *C. difficile*-derived MVs for 24 h compared to untreated cells (Figure 7A,B). In contrast, non-toxigenic *C. difficile*-derived MVs did not induce significant changes in FIS1, DRP1 and GPX1 mRNA expression compared to untreated cells (Figure 7A,B), but showed significant differences in FIS1 and DRP1 mRNA expression as compared with toxigenic *C. difficile*-derived MVs (Figure 7A).

### 3.7. Purified Toxigenic C. difficile-Derived MVs Do Not Affect Mitochondrial Biogenesis or Trigger Apoptosis in HepG2 Cells

To rule out that the effects of purified *C. difficile*-derived MVs on mitochondrial fission genes are related to mitochondrial biogenesis, we assessed the expression of PGC1A mRNA, the master gene of mitochondrial biogenesis. We found that toxigenic and non-toxigenic *C. difficile*-derived MVs did not alter the PGC1A mRNA expression in HepG2 cells (Figure 7C). Finally, we further explored the effect of purified *C. difficile*-derived MVs on apoptosis through CASP3 mRNA expression. We found that CASP3 expression was increased in HepG2 cells exposed to toxigenic *C. difficile*-derived MVs, but not in those exposed to non-toxigenic *C. difficile*-derived MVs, with significant differences between them (Figure 7D).

### 3.8. Glycolysis Key Enzymes Are Up-Regulated by Purified Toxigenic C. difficile-Derived MVs in HepG2 Cells

Due to the observed effects on mitochondrial function, we sought to explore the impact of purified *C. difficile*-derived MVs on glycolytic target genes. We only found a significant increase in HK2, LDHA, PDK1 and PKM2 expression (Figure 7E) in HepG2 cells treated with toxigenic *C. difficile*-derived MVs compared to untreated cells. Non-toxigenic *C. difficile*-derived MVs did not induce significant changes in mRNA expression, which was significantly decreased in LDHA and PKM2 compared to toxigenic *C. difficile*-derived MVs (Figure 7E).

### 3.9. Anti- and Pro-Inflammatory Interleukins Are Up-Regulated by Purified Toxigenic C. difficile-Derived MVs in HepG2 Cells

One of the key processes in the development of various liver diseases is hepatic inflammation. In this case, we have measured a pro-inflammatory (IL-6) and an anti-inflammatory (IL-10) interleukin. We found a significant increase in IL-6 and IL-10 expression (Figure 7F) in HepG2 cells treated with toxigenic *C. difficile*-derived MVs compared to untreated cells. Non-toxigenic *C. difficile*-derived MVs did not induce significant changes.

### 3.10. β-Oxidation, but Not Fatty Acid Synthesis, Is Up-Regulated by Purified C. difficile-Derived MVs in HepG2 Cells

We also wanted to analyze the effects of purified *C. difficile*-derived MVs on other key processes in the development of various liver diseases, such as the β-oxidation (CPT1A) and synthesis of fatty acid (FASN). We found that purified *C. difficile*-derived MVs significantly increased CPT1a expression in HepG2 cells, regardless of whether the MVs were from a toxigenic or non-toxigenic *C. difficile* strain (Figure 7G). However, FASN expression did not change with purified toxigenic or non-toxigenic *C. difficile*-derived MVs (Figure 7G).

## 4. Discussion

**Figure 8:**
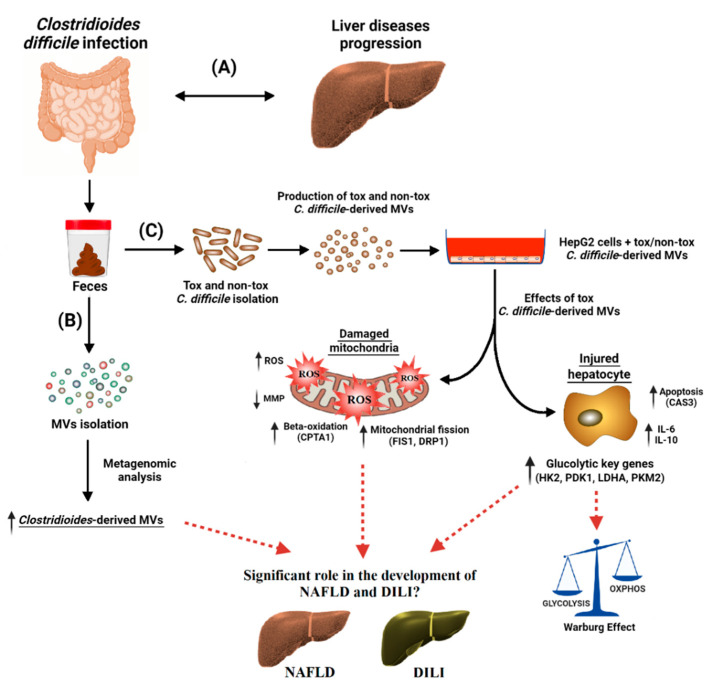
*Clostridioides difficile* infection (CDI) has been found to be associated with a broad range of liver diseases (A). Membrane vesicles (MVs) were isolated from the feces of CDI patients, and the microbiome profiling of these MVs was performed by metagenomic analysis. *Clostridioides*-derived MVs were present in a higher percentage (B). For the in vitro study (C), toxigenic (tox) and non-toxigenic (non-tox) *C. difficile* strains were isolated and cultured in the laboratory and the MVs that they produced were isolated. These *C. difficile*-derived MVs were internalized by HepG2 cells, with significant effects on mitochondrial function and gene expression. Toxigenic *C. difficile*-derived MVs decreased mitochondrial membrane potential (MMP) and increased intracellular reactive oxygen species (ROS). They also increased the expression of mitochondrial fission (FIS1 and DRP1), antioxidant (GPX1), apoptosis (CASP3), and beta-oxidation (CPT1A) genes, as well as pro- and anti-inflammatory genes (IL-6 and IL-10). In addition, glycolytic key genes (HK2, PDK1, LDHA and PKM2) were upregulated, possibly activating the Warburg effect in HepG2 cells. Non-toxigenic *C. difficile*-derived MVs, on the other hand, had no effect on gene expression except for an increase in CPT1A. These findings suggest that toxigenic *C. difficile*-derived MVs may play a significant role in the development and progression of different liver diseases, such as non-alcoholic fatty liver disease (NAFLD) and drug-induced liver injury (DILI). These liver diseases seem to share molecular mechanisms related to mitochondria. The mechanisms by which these MVs influence hepatic gene expression and mitochondrial function warrant further investigation, and may provide valuable insights into the treatment and prevention of these diseases.

EVs are nowadays considered an essential mechanism for bacterial and intercellular communication, as well as cargo carriers. Only recently has the composition of bacterial EVs found in feces been described. In the present study (see Figure 8), we have found for the first time a higher proportion of *C. difficile*-derived MVs in fecal EVs from patients with CDI than from those without CDI. In addition, we have shown in vitro that *C. difficile*-derived MVs from a toxigenic strain produced an increase in ROS generation, a disruption of MMP, and an up-regulation of mitochondria fission and apoptosis key genes (Figure 8). Moreover, we highlight a metabolic shift towards increased glycolytic metabolism and the increased expression of genes related to immune response and β-oxidation with toxigenic *C. difficile*-derived MVs (Figure 8).

In this study, we demonstrated that CDI induced an increase in EVs of *Clostridioides* and other opportunistic pathogens, and a decrease in EVs of bacteria protective against the development of liver damage, compared to those patients without CDI. Although the microbial diversity and richness did not differ between EVs obtained from feces of patients with and without CDI, evenness was higher in the case of CDI. In studies of gut microbiota, the analysis of bacterial diversity generally showed a reduction in CDI compared to control subjects, although some other studies found no difference in bacterial diversity and richness [38,39]. However, in our study, the second group consisted of patients with diarrhea due to causes other than *C. difficile*, rather than healthy control subjects, which may explain a reduction of evenness in these samples. EVs from fecal CDI samples showed a significant increase in *Clostridioides* and *Staphylococcus* genera. The increased proportion of EVs derived from these genera may reflect what is happening in the gut microbiota. Following *C. difficile* colonization and infection, inflamed epithelial cells and damaged mucosal tissue may be predisposed to colonization by other opportunistic pathogens such as *Staphylococcus aureus* (*S. aureus*) [40]. The intestinal coexistence of *C. difficile* and *S. aureus* has been previously described in patients with inflammatory bowel disease (IBD) [41], which may be related to antacid treatment, which increases co-colonization and overgrowth of opportunistic bacteria from the oral and nasal cavity [41]. In addition, we observed a reduction of the phylum Proteobacteria, the family *Christensenellaceae*, and the genera *Parasutterella* and *Ruminiclostridium* in fecal EVs from patients with CDI compared to those without CDI. A decrease of *Parasutterella* has previously been reported in patients with CDI and asymptomatic carriers compared to healthy donors [42]. Moreover, the abundance of *Parasutterella* has been reported to decrease in the gut microbiota of patients with NAFLD [3]. Another study describes the potential role of *Parasutterella* in the maintenance of bile acids and cholesterol metabolism [43]. The role of bile acids in the progression of NAFLD and other liver diseases has been previously recognized [44,45].

Although the relationship between microbiome and liver diseases may be due to a mixture of effects of different bacteria, we have focused on analyzing the effects of *C. difficile*-derived MVs, given a previous study in male C3H/HeN mice treated with *C. difficile* [21]. This study showed that intestinal *C. difficile* could initiate and aggravate liver injury. *C. difficile*-derived MVs carry pathogen-associated molecular patterns (PAMPs) and different protein and virulence factors that have detrimental effects on the host. PAMPs and lipopolysaccharides can act as costimulatory signals, enhancing the immune response and cytokine release in NAFLD and DILI [46,47]. In our work, we isolated the *C. difficile*-derived MVs to study their uptake into HepG2 cells, as well as their potential to modify the expression of several genes related to the development of NAFLD and/or DILI. Similar to what we observed, *C. difficile* has previously been found to secrete MVs during in vitro culture [8].

To elucidate whether or not *C. difficile*-derived MVs have any effects on hepatic cells, we first studied the internalization of *C. difficile*-derived MVs within the HepG2 cell. We found that *C. difficile*-derived MVs were successfully incorporated within HepG2 cells. Moreover, this uptake was independent of the strain of origin and the method used to isolate the MVs. However, we found that the effects of *C. difficile*-derived MVs depended on the strain of origin and the method used to isolate them. First, the effects produced by toxigenic *C. difficile*-derived MVs are clearly more significant and are different from those of non-toxigenic *C. difficile*-derived MVs at the level of morphological changes and the DAPI fluorescence intensity of HepG2 cells. A previous study found that toxigenic *C. difficile*-derived MVs trigger a pro-inflammatory response and encourage cytotoxicity in human colorectal epithelial Caco-2 cells [8]. However, the method of MVs isolation is also of great importance. We found that toxigenic *C. difficile*-derived MVs isolated by the ultracentrifugation method alone produced the greatest morphological changes and decreases in the DAPI fluorescence intensity in HepG2 cells, which is associated with a high cytotoxic effect and cell death rate [36,37]. This may be due to the presence of different molecules, together with the MVs present in the *C. difficile* culture in particular toxins that could not be fully separated from the MVs in their isolation by ultracentrifugation. *C. difficile* toxins are known to induce F-actin depolymerization and reorganization of the actin cytoskeleton [48], and cell death [49]. Together, toxins and MVs may be responsible for this enhanced cytotoxic effect on HepG2 cells. For this reason, in this work we sought to explore the role of these MVs on hepatic cells, independently of the effect of toxins, in order to gain knowledge about the possible mechanisms linking gut microbiota with hepatic injury. To this end, we added an SEC purification step in the isolation of *C. difficile*-derived MVs. As we have demonstrated with this step, we have been able to separate *C. difficile*-derived MVs from the presence of soluble protein impurities as toxins. This purification tool provides a better recovery of pure MVs [26].

In a subsequent step, and due to the possible link found in the literature between CDI and liver disorders [20,50,51], we studied the possible impact of purified *C. difficile*-derived MVs on HepG2 cells’ metabolism, depending on whether the *C. difficile* strain was toxigenic or not. By internalizing the *C. difficile*-derived MVs within HepG2, their cargo of functionally active molecules can be released and can regulate gene expression and modify specific signaling pathways, as shown in other studies [8]. Firstly, we focused on the study of mitochondrial function. Mitochondria have been considered an important target in NAFLD and DILI [46,47]. Although we observed a decrease in MMP after incubation with toxigenic and non-toxigenic *C. difficile*-derived MVs, only those MVs from toxigenic *C. difficile* resulted in increased cellular stress through intracellular ROS generation. This mechanism of action, the elevation of intracellular ROS and loss of MMP, has already been described for other molecules [52]. The decrease found in MMP may imply that the process that damages the mitochondria exceeds the capacity to fix/remove the damaged ones [53] and could trigger a mitochondrial degradation [54]. In line with our results, it has been reported that porin B present in outer membrane vesicles (OMVs) of *Neisseria meningitidis* induced a loss of MMP in macrophages [55]. In addition, the increase found in ROS would be playing as anti-microbial molecules, by recognizing bacterial components present in MVs, as it contributes to pathogen clearance during infections [56]. In this regard, an oxidative stress imbalance (excessive generation of ROS) has been proposed as a major pathogenic factor in the development of NAFLD, non-alcoholic steatohepatitis (NASH), cirrhosis and DILI [22,46,47]. Meanwhile, the increase in GPX1 produced by toxigenic *C. difficile*-derived MVs would be compatible with an antimicrobial response driven by cellular antioxidant systems that regulate redox homeostasis by counteracting the increased ROS production. GPX1 is the main GPX in the mammalian liver, and plays a significant role in ameliorating ROS damage [57]. Taken together, we hypothesize that although *C. difficile*-derived MVs can alter MMP, only those derived from toxigenic *C. difficile* may play a key role in mitochondrial function in the context of liver injury.

Increased ROS levels also have the capacity to promote the expression of inflammatory cytokines [46], a feature also present in the liver of diseases such as NAFLD and DILI. In agreement with this, we found that toxigenic *C. difficile*-derived MVs are able to increase the expression of IL-6, a pro-inflammatory cytokine. Moreover, they also increase the expression of IL-10, an anti-inflammatory cytokine, possibly to counteract the increased inflammatory state produced by this type of MVs. The current results indicate an increased expression of both interleukins in HepG2 cells, which are cancer cells that may already have altered IL-6 and IL-10 expression themselves. Therefore, our results may not be reproducible in non-cancerous liver cells. In this regard, further studies are needed to better understand the pathological mechanism underlying the toxic effect of *C. difficile*-derived MVs on primary liver cells.

We have also analyzed the effect of *C. difficile*-derived MVs on fatty acids’ β-oxidation. CPT1A is the rate-limiting enzyme of β-oxidation in the liver. This mitochondrial process, under normal conditions, results in energy production. However, when this process becomes excessive, as occurs in NAFLD, fatty acids oxidation increases at other sites, including peroxisomes and microsomes, thereby increasing ROS production. In our study, we found an increase in the expression of CPT1A with MVs derived from toxigenic and non-toxigenic *C. difficile* strains. This finding could also associate *C. difficile*-derived MVs with the development of NAFLD and NASH. Recently, it has been reported that CPT1A was overexpressed in hepatic stellate cells from patients with liver fibrosis, correlating positively with fibrosis and NAFLD activity score [58]. Furthermore, the specific deletion of CPT1A in mouse hepatic stellate cells protected against fibrosis [58]. On the other hand, the liver of an animal model with deficient CPT1A function likely represents a “healthy” fatty liver that fights against the deleterious actions of the high-fat diet on hepatic injury [59]. In the early stages of fatty liver, in which the liver is otherwise healthy, the liver compensates by decreasing CPT1A activity [59]. All this suggests that the increase of CPT1A expression could be increasing β-oxidation, with the consequent increase in ROS production found, as suggested by another study. During NAFLD progression, an increase in mitochondrial β-oxidation could lead to an overproduction of ROS [60].

When hepatocytes are exposed to high levels of cellular stress, like in NASH, the mitochondrial network becomes more fragmented (fission), loses its MMP, and increases ROS [53,61,62]. Fission is essential for mitochondrial degradation. Regarding the impact of *C. difficile*-derived MVs on this process, we highlight here the up-regulation of key mitochondrial fission genes (FIS1 and DRP1) triggered only by toxigenic *C. difficile*-derived MVs. Accumulating evidence suggests that bacteria can manipulate the mitochondrial network. Previous studies have shown that LPS-stimulated macrophages harbor shortened (fission profile) mitochondria via the activation of DRP1 and ROS production [63], conditions that we also found in our study with toxigenic *C. difficile*-derived MVs. In addition, the *L. pneumophila* T4SS effector MitF (also called LegG1) induced a DRP1-dependent mitochondrial fragmentation [64]. This increased mitochondrial fission may be involved in the development of the NAFLD spectrum and DILI, as suggested by several studies [65,66]. Although the mitochondrial fission profile is increased, toxigenic *C. difficile*-derived MVs slightly, but not significantly, decreased PGC1A mRNA expression, corroborating that mitochondrial biogenesis would not be modified after the treatment with these MVs. This process, mitochondrial biogenesis, has been found to be slightly decreased in obese individuals with NAFLD and markedly decreased in those with NASH [67].

Recent studies have found that the modification of fission mitochondrial dynamics deregulates cell metabolism [61]. In particular, the fission process is associated with a glycolytic metabolic profile [68,69]. In this context, we found that glycolysis-limiting enzymes, such as HK and PKM2, increased after treatment with toxigenic *C. difficile*-derived MVs, thus shifting the metabolic balance in HepG2 towards glycolysis and reinforcing the Warburg effect. This effect is characterized by increased aerobic glycolysis and lactate production, and is associated with NAFLD and liver cirrhosis [70,71]. PKM2 is activated to increase glycolysis in LPS-stimulated macrophages [72]. In addition, PKM2 can translocate to the cell nucleus to induce the expression of glycolytic enzymes (e.g., LDHA) [73] and play a pro-apoptotic role [74]. In our study, metabolic changes similar to those found in fatty liver hepatocytes have been observed. Moreover, adenoviral-mediated overexpression of HK2 and PKM2 promoted liver steatosis [75]. Likewise, the upregulation of PKM2 is consistent with the PKM2 activation found in NASH [76]. Taken together, the increased expression of glycolytic enzymes produced by toxigenic *C. difficile*-derived MVs appears to be associated with the development of fatty liver.

Pyruvate, a product of glycolysis, can be transformed to acetyl-coenzyme A by the pyruvate dehydrogenase complex in mitochondria, whose activity is regulated by PDK1-4, or into lactate by LDHA. In the present study, we have found that the expression of these two enzymes increased in HepG2 cells after treatment with toxigenic *C. difficile*-derived MVs, suggesting an increase in aerobic glycolysis and lactate production (Warburg effect). Lactate production in fatty liver is known to be significantly higher than in normal liver [77,78], which worsens hepatic steatosis and increases the expression of genes involved in lipogenesis and fatty acids uptake [78].

Another pathogen-induced process associated with mitochondrial fission is the reduced efficiency of endoplasmic reticulum Ca^2+^ uptake, a key effector in apoptotic signaling [64]. Apoptosis is a landmark in the progression from NAFLD to NASH. In relation to this effect, here we found an increased level of CASP3 mRNA level, a marker of apoptosis. Its increase could be associated with increased mitochondrial fission, or due to the excessive production of ROS, which attack the intracellular macromolecular compound and lead to cell death or induce apoptosis [79]. Both of these events occurred when HepG2 cells were incubated with toxigenic *C. difficile*-derived MVs. Our findings would be consistent with previous results with other OMVs. Macrophages exposed to OMVs from *Neisseria meningitidis*, enterohemorrhagic, *Escherichia coli*, and *Pseudomonas aeruginosa* induced mitochondrial apoptosis [55].

This study has several limitations. On the one hand, HepG2 cells were collected only to analyze mRNA expression, so we cannot measure the products of these enzymatic pathways. On the other hand, the results of this study are obtained from HepG2 cells, a carcinoma cell line. Although it could be a good first step in the right direction, further studies are needed to evaluate whether toxigenic *C. difficile*-derived MVs can also produce these changes in primary human hepatocytes to provide a more accurate interpretation of our results. Furthermore, to our knowledge, there is no study that has measured the presence of *C. difficile*-derived MVs in the blood. Therefore, we do not know whether the amount of MVs used in our study are clinically relevant, but it may give an idea of the effects that may be occurring in the liver of patients with acute CDI.

In conclusion, the present study demonstrated that there is an increase of *C. difficile*-derived MVs in CDI. Furthermore, we showed different mechanisms of liver injury orchestrated by *C. difficile*-derived MVs in HepG2 cells. We have identified for the first time a different pattern of metabolic and mitochondrial damage produced by *C. difficile*-derived MVs depending on the *C. difficile* strain. The effects detected in HepG2 cells after incubation with toxigenic *C. difficile*-derived MVs are common pathophysiologic features observed in different liver diseases, such as NAFLD spectrum and DILI. The results of this study reveal the close relationship in the gut–liver axis and increase knowledge of the association between gut microbiota and liver diseases. Further in-depth studies with a combination of different bacterial EVs are needed in order to identify the molecular mechanisms underlying the pathophysiology of microbiota-induced liver injury, as well as to elucidate possible concomitant treatments for these liver diseases.

## Figures and Tables

**Table 1:** List of primers.

**Gene Symbol**	**Gene ID** **(ENSEMBL)**	**Sequence (5′-3′)**	**TM (°C)**
DRP1	ENSG00000087470	forward: GTGAACCCGTGGATGATAAA reverse: GAAACCTCAGGCACAAATAAAG	62
FIS1	ENSG00000214253	forward: AGCGGGATTACGTCTTCTA reverse: CCACGAGTCCATCTTTCTTC	60
GPX1	ENSG00000233276	forward: CCCTGCGGGGCAAGGTACTA reverse: GGGCATCAGGAGAACGCCAA	60
LDHA	ENSG00000134333	forward: TATGGAGTGGAATGAATGTTG reverse: CCCTTAATCATGGTGGAAACT	60
HK2	ENSG00000159399	forward: TAGCCTTCTTTGTGCGCCGT reverse: GGTCAACCTTCTGCACTTGGTCA	69
PDK1	ENSG00000152256	forward: TGCCTCTGGCTGGTTTTGGTTAT reverse: TGTCTAGGCACTGCGGAACG	68
PKM2	ENSG00000067225	forward: ACTCACTCTGGGCTGTAA reverse: CCTCCTTCTTCCCTTGATTG	60
PGC1A	ENSG00000109819	forward: CACCAGCCAACACTCAGCTA reverse: GTGTGAGGAGGGTCATCGTT	62
CASP3	ENSG00000164305	forward: TGCCCTGACTTCTCTGTAGC reverse: TTGGAGCCACACAGACCTAG	60
CPT1A	ENSG00000110090	forward: CAGCATATGTATCGCCTCGC reverse: CTGGACACGTACTCTGGGTT	60
FASN	ENSG00000169710	forward: CCCTCATCTCCCCACTCATC reverse: CAGCGTCTTCCACACTATGC	60
IL-6	ENSG00000136244	forward: CACACAGACAGCCACTCACC reverse: TTTTCTGCCAGTGCCTCTTT	62
IL-10	ENSG00000136634	forward: GCTCTGTTGCCTGGTCCTC reverse: TGTCTGGGTCTTGGTTCTCA	63
β-actin	ENSG00000075624	forward: TACAGCTTCACCACCACGGC reverse: AAGGAAGGCTGGAAGAGTGC	64

DRP1: dynamin-related protein. FIS1: mitochondrial fission 1 fission. GPX1: glutathione peroxidase 1. LDHA: lactate dehydrogenase A. HK2: hexokinase 2. PDK1: pyruvate dehydrogenase kinase 1. PKM2: pyruvate kinase M2. PGC1A: peroxisome proliferator-activated receptor γ co-activator 1 α. CASP3: caspase 3. CPT1A: carnitine palmitoyltransferase 1A. FASN: fatty acid synthase. IL-6: interleukin-6. IL-10: interleukin-10.

## Data Availability

The data that support the findings of this study are deposited in the Bioproject (National Center for Biotechnology Information (NCBI)) at http://www.ncbi.nlm.nih.gov/bioproject/945623 (accessed on 17 March 2023), reference number [PRJNA945623]. The data that support the findings of this study are available from the corresponding author, [EGF], upon reasonable request.
